# The Association between the History of Cardiovascular Diseases and Chronic Low Back Pain in South Koreans: A Cross-Sectional Study

**DOI:** 10.1371/journal.pone.0093671

**Published:** 2014-04-21

**Authors:** In-Hyuk Ha, Jinho Lee, Me-riong Kim, Hyejin Kim, Joon-Shik Shin

**Affiliations:** 1 Jaseng Medical Foundation, Jaseng Hospital of Korean Medicine, Seoul, Republic of Korea; 2 Graduate School of Public Health & Institute of Health and Environment, Seoul National University, Gwanak-ro, Gwanak-gu, Seoul, Korea; 3 Department of Herbology, Graduate School of Korean Medicine, Kyung Hee University, Seoul, Republic of Korea; University of Louisville, United States of America

## Abstract

**Background:**

Cardiovascular disease and related risk factors have been suggested as a mechanism leading to atherosclerosis of the lumbar vessels and consequent lumbar pain or sciatica. But there is continued controversy concerning its generalization. This study examined whether cardiovascular disease or its risk factors were associated with chronic low back pain (cLBP) in Koreans.

**Methods:**

Health surveys and examinations were conducted on a nationally representative sample (n = 23,632) of Koreans. A total of 13,841 eligible participants (aged 20 to 89 years) were examined to determine the association between cardiovascular disease, the Framingham risk score, major cardiovascular risk factors (blood pressure, diabetes, cholesterol, and smoking habits) and chronic LBP.

**Results:**

The total prevalence of cLBP was 16.6% (men: 10.8%, women: 21.1%) and that in patients with a history of cardiovascular diseases was 36.6% (men: 26.5%, women: 47.1%). The results showed that patients’ medical history of cardiovascular disease was significantly associated with cLBP in both men and women when adjusted for covariates (men: OR 2.16; 95%CI 1.34∼3.49; women: OR 2.26; 95%CI 1.51∼3.38). No association was observed between cLBP and the Framingham risk score, medication for hyperlipemia, hypertension, diabetes, and major cardiovascular risk factors (systolic blood pressure, total cholesterol, high density lipoprotein cholesterol, triglycerides, glucose and smoking habits) in either men or women.

**Conclusions:**

The prevalence of cLBP is correlated to a history of cardiovascular disease, but not to the major cardiovascular risk factors from the Framingham study. Further studies on whether these results were affected by psychological factors in patients with a history of cardiovascular diseases or whether new potential risk factors from the artery atherosclerosis hypothesis applying to Koreans exist are needed.

## Introduction

Low back pain (LBP) is a common disorder which negatively impacts both individual and society. 70 to 80% of adults suffer from low back pain at some point in their lives [1]. Approximately 6 to 10% patients who initially suffer from acute LBP develop chronic LBP or experience repeated fluctuating pain episodes, and health care services are needed to counteract symptoms which in turn incur substantial social costs [2]. LBP is the fifth most frequent reason for clinic visits in the United States [3]. In addition, indirect costs related to work loss are also substantial with many patients reporting dysfunction and disruption of daily activities and work due to pain, and 2% of the U.S. work-force are compensated for back injuries each year [4]. Therefore, a more accurate and efficient screening system for risk factors of chronic LBP seems important for better understanding and prevention of the transition to chronic pain and disability.

There have been many studies aiming to determine the cause of LBP, and it has usually been accepted that biomechanical loading of the spine [5], smoking [6], psychosocial influences [7], obesity [8], and BMI [9], [10] are causes of low back pain. Studies on whether cardiovascular disease and its risk factors could be a cause of low back pain initially began with the evaluation of 56 postmortem lumbar aortograms. Eighty-eight percent of subjects with back pain history had one or more missing lumbar arteries which was high when compared with the 59% of age-matched controls [11]. Atherosclerosis of the lumbar arteries has received continued attention as a possible underlying factor for back disorders as ischemia of the lumbar spine can cause damage or degeneration of the main structure causing pain [12]. Studies have also been conducted not only on atherosclerosis, but also on the association of serum lipids and blood pressure, which can be the cause of atherosclerosis and perhaps back pain [1].

The Framingham Heart Study introduced the concept of risk factors in cardiovascular diseases by identifying common factors that contribute to cardiovascular disease by following its development over 50 years [13]. The Framingham risk score estimates the 10 year risk of cardiovascular disease by assessing the following factors which affect cardiovascular disease – gender, age, systolic blood pressure (SBP), use of hypertension treatment, smoking habits, diabetes mellitus, total cholesterol, and high density lipoprotein(HDL) cholesterol [14]. Therefore, the Framingham risk score could be useful in examining the association between cardiovascular risk factors and chronic LBP.

The present study aimed to find out whether the Framingham risk score and main risk factors of cardiovascular diseases such as blood pressure, diabetes, cholesterol and smoking habits, as determined by the Framingham study were associated with chronic LBP. The possible confounding effects of socioeconomic status (education, income and occupation), lifestyle risk factors (alcohol drinking and exercise habits) and BMI, which is closely related to serum lipid, were considered in the analysis. The study sample was representative of the Korean adult population.

## Methods

### 2.1. Study Population and Sampling

This study was based on data obtained from the Korean National Health and Nutritional Examination Survey (KNHANES) IV (2007–2009), which used a rolling sampling design that involved a complex, stratified, multistage, probability-cluster survey of a representative sample of the non-institutionalized civilian population of South Korea. The survey was performed by the Korean Ministry of Health and Welfare and had three components: the health survey, health examination, and nutrition survey. Further information can be found in “The 4^th^ (2007–2009) KNHANES Sample Design” and the 1^st^∼3^rd^ Sample Design reports, which are available on the KNHANES website [15]. The data from KHANES is available on request by email if the applicant logs onto the ‘Korea National Health and Nutrition Examination Survey’ website and specifies which annual reports he or she needs. The KNHANES IV (2007–2009) examination and health survey was completed by 23,632 subjects (74.5% of the total target population of 31,705). The present analysis was confined to 13,841 respondents aged 20 to 89 years who answered the back pain examination survey and had no missing values of covariance or cardiovascular risk factors.

### 2.2. Chronic Low Back Pain

Chronic LBP patients were defined as individuals who reported episodes of back pain lasting three months or longer during the previous year in the health survey.

### 2.3. Cardiovascular Disease Patients, Cerebrovascular Disease Patients

Cardiovascular disease patients were defined as individuals who reported having suffered from cardiac infarction or angina. Cerebrovascular disease patients were defined as individuals who reported having experienced ischemic stroke(s).

### 2.4. The Framingham Risk Score

Of the 13,841 total target population, individuals with cardiovascular or cerebrovascular disease history were excluded, and 13,299 patients were assessed for the association between Framingham risk scores, hyperlipemia, hypertension, and diabetes medication, and chronic LBP. The Framingham risk score was calculated through an algorithm using the serum total cholesterol(TChol) level. This algorithm divided the subjects in 9 groups by age and further divided them into 5 subgroups within 4 categories(total cholesterol level, high-density lipoprotein cholesterol level, systolic blood pressure, diastolic blood pressure). We assigned risk values to each group according to their gender. The subjects were categorized into subgroups by whether or not they currently smoked or had diabetes, and again assigned risk values according to their gender. The Framingham risk score was calculated as the sum of the values resulting from these 6 steps. Conclusively, the possibility of risk factors with regard to the calculator prepared by R.B. D’Agostino et al. [16] was obtained and the outcomes were classified into five groups according to risk. The group with the highest risk factor was compared to the other groups.

### 2.5. Hyperlipemia, Hypertension and Diabetes

In the Seventh Joint National Committee on the Prevention, Detection, Evaluation, and Treatment of High Blood Pressure (JNC 7), prehypertension was defined to be systolic blood pressure (SBP) in the range of 120 mmgHg to 139 mmgHg, or diastolic blood pressure (DBP) from 80 mmHg to 89 mmHg. Hypertension was defined when SBP was above 140 mmHg, DBP was above 90 mmHg or when taking medication for blood pressure control. Following National Cholesterol Education Program Adults Treatment Panel III(NCEP ATPIII) [17], hypercholesterolemia was defined when TChol was above 240 mg/dl or when patients were already on medication for control of hypercholesterolemia. Triglycerides(TG) above 200 mg/dl was defined as hyperlipidemia and HDL below 40 mg/dl was defined as low HDL cholestrolemia. According to the American Diabetes Association, impaired fasting glucose was defined as a fasting glucose level between 110 mg/dL to 125 mg/dL. Fasting glucose level above 126 mg/dl or those on insulin medication treatment were defined as diabetes.

### 2.6. Cardiovascular Risk Factors

We investigated the cardiovascular risk factors of 10,592 people who had no history of cardio-cerebrovascular disease and no history of medication taken for hyperlipemia, hypertension, or diabetes. Smoking status was divided into three categories; current smokers, past smokers and nonsmokers. Systolic and diastolic pressure are presented as the mean value of the second and third blood pressure measurements out of three independent measurements. Serum concentrations of TChol, HDL cholesterol, TG, and glucose after a minimum 8 hour fast were analyzed. For BP analyses, the upper half of the group(>148 mmHg) with systolic blood pressure above 140 mmHg was compared to the prehypertension(120–138 mmHg) and normal blood pressure groups(≤119 mmHg), and the upper half of the group(>95 mmHg) with diastolic blood pressure above 90 mmHg was compared to the prehypertension(80–89 mmHg) and normal groups(≤79 mmHg). In analyzing total cholesterol and TG, the upper half of the groups with values above normal range(cholesterol≥255 mg/dl, triglycerides≥255 mg/dl) were compared to the upper(cholesterol 183–239 mg/dl, triglycerides≥255 mg/dl) and lower halves of the normal range groups(cholesterol≤182, triglycerides≤102). The lower half(≤35 mg/dl) of patients in the hypoalphalipoproteinemia group was compared to the upper(≥51 mg/dl) and lower halves(40–50 mg/dl) of the normal range group(≥40 mg/dl). For glucose, the diabetic group(≥126 mg/dl), impaired fast glucose group(110–125 mg/dl) and normal group(≤109 mg/dl) were compared.

All blood analyses were carried out by Seoul Institution of Medicine and Science and Neodin Medical Institute (NMI) which are both institutions certified by the Korean Ministry of Health and Welfare.

### 2.7. Covariates

The gender, age, socioeconomic status, and lifestyle of subjects were analyzed. Education was classified as ≤6, 7–9, 10–12 or >13 years of formal education. Households were broken into income quartiles by gender and age according to the average monthly household income adjusted for family size using the equivalence scale (monthly household income/number of household members). Occupation classification was further divided from the Korean Standard Classification of Occupation, 6th revision into 7 groups; (a) administrator, manager, or professional practitioner, (b) office worker, (c) service or retail industry worker, (d) agriculture or fishery industry worker, (e) equipment mechanic or machinery operator, (f) manual worker, and (g) unemployed. BMI (kg/m^2^) was categorized into 3 brackets according to physical measurements (<18.5, 18.5–24.9, 25≤). Alcohol consumption was divided into two categories; nondrinkers (those with no alcohol consumption during the past year or whose frequency of alcohol intake was less than once a month) and drinkers (those with alcohol consumption at least once a month). Regular exercise patterns were sorted according to whether subjects did heavy exercise (running, climbing, fast biking, etc.) at least 20 minutes/session, 3 times/week, light exercise (swimming, tennis doubles, volleyball, etc.) at least 30 minutes/session, 5 times/week, or walked at least 30 minutes/session, 5 times/week during the past week.

### 2.8. Statistical Analysis

Logistic regression analysis was used to estimate associations between cardiovascular diseases, the Framingham risk score, hypertension, hypercholesterolemia, hypertriglyceridemia, hypoalphalipoproteinemia, diabetes, medication, serum lipid levels, blood glucose levels, blood pressure, smoking and low back pain with adjustments made for covariates. The statistical packages for Social Science for Windows, version 11.0 (SPSS Inc., Chicago, IL, U.S.A.) were used. Population weights were applied to the analyses to correct the distributions of the cluster sample regarding Primary Sampling Unit, covariance, and significance to correspond with those of the Korean population.

### 2.9. Ethics Statement

The interviewer was not given any prior information about a participant before he/she performed the interview, and all participants provided written consent to participate in the survey. The study protocol was approved by the Institutional Review Board of Jaseng Hospital of Korean Medicine in Seoul, Korea.

## Results

The total prevalence of chronic LBP was 16.6% (10.8% in men and 21.1% in women) and that of chronic LBP in patients who suffered from cardiovascular diseases was 36.6% (26.5% in men and 47.1% in women). In both men and women, the prevalence of chronic LBP was higher in subjects with a history of cardiovascular diseases than those with no history of cardiovascular diseases(Fully adjusted OR was 2.16 in men and 2.26 in women) (Table 1). The average and standard deviation of age, blood pressure, total cholesterol, HDL, TG, glucose levels and Framingham risk scores in patients who had no history of cerebro-cardiovascular diseases are listed by gender in Table 2. The covariates used were education, household income, occupation, BMI, alcohol drinking and exercise habits. Table 3 shows the age-adjusted relationships of covariates with chronic LBP.

**Table 1:** Associations by cardiovascular and cerebrovascular disease with chronic low back pain in Koreans aged 20 to 89 years.

		n (cases)	Adjusted for age		Fully adjusted^a^	
			OR	95% CI	OR	95% CI
**Cardiovascular diseases**						
Men	No	5925(618)	1.00		1.00	
	Yes	147(39)	2.09	1.32–3.30	2.16	1.34–3.49
Women	No	7629(1571)	1.00		1.00	
	Yes	140(66)	2.43	1.64–3.60	2.26	1.51–3.38
**Cerebrovascular diseases**						
Men	No	5934(622)	1.00		1.00	
	Yes	138(35)	1.74	1.02–2.97	1.53	0.91–2.58
Women	No	7631(1566)	1.00		1.00	
	Yes	138(71)	2.14	1.41–3.24	1.95	1.28–2.97

Logistic regression analysis. Odds ratios (OR) and 95% confidence intervals (CI).

a Adjusted for age, education, household income, occupation, BMI, alcohol drinking and exercise habits.

**Table 2:** Descriptive statistics of cardiovascular risk factors a.

	Men	Women
	mean(S.D.)	mean(S.D.)
**Age(years)**	47.8 (15.4)	48.3 (16.0)
**Systolic blood pressure(mmHg)**	120.7 (15.9)	115.5 (18.0)
**Diastolic blood pressure(mmHg)**	79.6 (10.6)	74.1 (10.5)
**Total cholesterol(mg/dl)**	187.0 (34.7)	187.5 (35.7)
**HDL- cholesterol(mg/dl)**	47.9 (12.0)	53.3 (12.7)
**Triglycerides(mg/dl)**	156.7 (126.2)	114.1 (79.4)
**Glucose(mg/dl)**	99.1 (24.0)	96.1 (22.9)
**Framingham risk score(%)**	12.8 (12.9)	10.8 (13.0)
**Medication of hypertension** b	14.1%	16.3%
**Medication of hyperlipemia** b	2.1%	3.5%
**Medication of diabetes** b	5.3%	5.1%

a Subjects with no history of cardiovascular disease or cerebrovascular disease.

b Percentage of patients on medication for hypertension, hyperlipidemia or diabetes in total subjects.

**Table 3:** Associations by background factors with age-adjusted odds ratios (OR) and 95% confidence intervals (CI) for chronic low back pain among Koreans aged 20 to 89 years^a^.

	Men			Women		
	n(case)	OR	95% CI	n(case)	OR	95% CI
**Household income**						
Low	972(168)	1.00		1583(541)	1.00	
Fairly low	1416(150)	0.88	0.66–1.17	1880(371)	0.78	0.64–0.94
Fairly high	1653(148)	0.84	0.61–1.16	1999(311)	0.75	0.61–0.92
High	1757(123)	0.69	0.50–0.95	2039(281)	0.69	0.55–0.86
**Education(years)**						
≤6	1060(212)	1.00		2442(541)	1.00	
7–9	712(104)	0.95	0.70–1.28	788(371)	0.71	0.56–0.90
10–12	2124(174)	0.72	0.53–0.99	2524(311)	0.58	0.45–0.74
>13	1902(99)	0.52	0.37–0.74	1747(281)	0.49	0.37–0.65
**Occupation**						
Administrator, manager, or professional practitioner	946(52)	1.00		748(61)	1.00	
Office worker	599(23)	0.58	0.33–1.02	493(31)	0.66	0.40–1.09
Service or retail industry worker	723(34)	0.75	0.46–1.22	1073(155)	1.23	0.87–1.74
Agriculture or fishery industry worker	707(163)	2.76	1.77–4.32	701(296)	3.07	2.01–4.67
Equipment mechanic or machinery operator	1181(101)	1.25	0.86–1.81	207(33)	1.63	0.87–3.06
Manual worker	492(54)	1.25	0.78–2.01	766(149)	1.17	0.80–1.72
Unemployed(student, housewife, etc.)	1150(162)	1.78	1.18–2.67	3513(779)	1.41	1.00–1.99
**Regular exercise**						
No	2346(213)	1.00		3402(623)	1.00	
Yes	3452(376)	1.07	0.84–1.35	4099(881)	1.28	1.11–1.48
**Alcohol drinking**						
No	1471(201)	1.00		4532(1071)	1.00	
At least once a month	4327(388)	0.85	0.67–1.07	2969(433)	0.88	0.75–1.04
**BMI(kg/m^2^)**						
<18.5,	205(30)	1.00		403(50)	1.00	
18.5–24.9	3558(379)	0.78	0.46–1.32	4965(929)	1.15	0.80–1.65
25≤	2035(180)	0.75	0.43–1.29	2133(525)	1.26	0.86–1.84

Logistic regression analysis.

a Subjects with no history of cardiovascular disease or cerebrovascular disease.

There were no statistically significant differences between the groups in the Framingham risk scores. In addition, there was no significant association between hyperlipidemia, hypertriglyceridemia, hypoalphalipoproteinemia, and diabetes with chronic LBP. The prevalence of chronic LBP was significantly lower in men who had hypertension compared to those who didn’t, but no associations were seen among women (Table 4).

**Table 4:** Associations by Framingham risk score and cardiovascular risk factors with chronic low back pain in Koreans aged 20 to 89 years^a^.

	Men	Adjusted for age		Fully adjusted^c^		Women	Adjusted for age		Fully adjusted^c^	
	n(case)	OR	95% CI	OR	95% CI	n(case)	OR	95% CI	OR	95% CI
**Framingham risk score** b										
High	1159(219)	1.00		1.00		1500(603)	1.00		1.00	
Middle	2320(235)	0.77	0.56–1.05	0.89	0.65–1.21	3001(607)	0.84	0.68–1.03	0.89	0.72–1.09
Low	2319(135)	0.93	0.57–1.51	1.11	0.65–1.91	3000(294)	1.00	0.71–1.42	1.25	0.86–1.82
**Hypertension**										
Normal	2152(224)	1.00		1.00		4122(607)	1.00		1.00	
Prehypertension	1820(169)	0.89	0.68–1.16	0.90	0.68–1.18	1489(305)	0.93	0.78–1.13	0.89	0.73–1.08
Hypertension	1826(196)	0.63	0.48–0.83	0.63	0.47–0.85	1890(592)	0.97	0.80–1.17	0.92	0.75–1.11
**Hypercholesterolemia**										
No	5272(538)	1.00		1.00		6643(1273)	1.00		1.00	
Yes	526(51)	0.77	0.53–1.12	0.80	0.55–1.18	858(231)	0.91	0.74–1.12	0.92	0.74–1.13
**Hypertriglyceridemia**										
No	4490(469)	1.00		1.00		6722(1277)	1.00		1.00	
Yes	1308(120)	0.88	0.69–1.12	0.90	0.70–1.16	779(227)	1.15	0.94–1.41	1.10	0.90–1.35
**Hypoalphalipoproteinemia**										
No	3822(382)	1.00		1.00		6040(1121)	1.00		1.00	
Yes	1976(207)	0.97	0.78–1.20	0.98	0.78–1.24	1461(383)	1.13	0.96–1.32	1.07	0.91–1.25
**Diabetes**										
Normal	3891(373)	1.00		1.00		5723(1048)	1.00		1.00	
Impaired Fasting Glucose	1340(138)	0.87	0.69–1.10	0.90	0.71–1.15	1179(278)	0.89	0.74–1.07	0.87	0.72–1.05
Diabetes	567(78)	0.94	0.68–1.29	0.94	0.67–1.30	599(178)	0.86	0.68–1.08	0.82	0.65–1.03

Logistic regression analysis. Odds ratios (OR) and 95% confidence intervals (CI).

a Indicates subjects with no history of cardiovascular or cerebrovascular diseases. Diagnosis was made when patients met international standards or were already on medications for associated diseases.

b High indicates the uppermost quintile of the highest, middle 2^nd^ and 3^rd^ quintiles and lowest two quintiles.

c Adjusted for age, education, household income, occupation, BMI, alcohol drinking and exercise habits.

Diastolic pressure was associated with chronic LBP in both men and women, and glucose was found to be associated in women (Table 5).

**Table 5:** Associations by cardiovascular risk factors with chronic low back pain among Koreans aged 20 to 89 years^a^.

	Men	Adjusted for age		Fully adjusted^c^		Women	Adjusted for age		Fully adjusted^c^	
	n(case)	OR	95% CI	OR	95% CI	n(case)	OR	95% CI	OR	95% CI
**Systolic blood pressure(mmHg)** b										
Hypertension (≥148)	224(32)	1.00		1.00		203(49)	1.00		1.00	
Hypertention((140–148)	218(22)	0.86	0.43–1.69	0.85	0.42–1.71	209(62)	1.39	0.82–2.35	1.33	0.78–2.26
Pre-hypertension (120–139)	1585(156)	1.22	0.74–2.02	1.29	0.78–2.13	1153(267)	1.54	0.99–2.39	1.46	0.94–2.28
Normal (≤119)	2767(250)	1.30	0.81–2.08	1.44	0.90–2.31	4462(628)	1.40	0.90–2.17	1.43	0.92–2.22
**Diastolic blood pressure(mmHg)** b										
Hypertension (≥95**^)^**	360(22)	1.00		1.00		200(35)	1.00		1.00	
Hypertension(90–95)	466(37)	1.48	0.81–2.69	1.54	0.84–2.80	220(45)	1.43	0.79–2.57	1.48	0.81–2.68
Prehypertension (80–89)	1514(122)	1.68	0.95–2.97	1.76	0.99–3.12	1092(217)	1.47	0.94–2.31	1.56	1.00–2.44
Normal (≤79)	2454(279)	2.12	1.23–3.63	2.15	1.24–3.75	4515(709)	1.61	1.06–2.45	1.71	1.12–2.62
**Total cholesterol(mg/dl)** b										
Hypercholesterolemia (≥255)	169(15)	1.00		1.00		212(43)	1.00		1.00	
Hypercholesterolemia(240–255)	185(16)	1.13	0.49–2.61	1.23	0.53–2.86	213(40)	0.95	0.54–1.67	0.92	0.51–1.66
Upper normal (183–239)	2207(204)	1.37	0.72–2.61	1.47	0.77–2.81	2813(538)	1.27	0.85–1.90	1.22	0.81–1.84
Lower normal (≤182)	2233(225)	1.57	0.83–2.96	1.64	0.87–3.10	2789(385)	1.31	0.88–1.97	1.29	0.85–1.96
**Triglycerides(mg/dl)** b										
Hypertriglyceridemia (≥268)	511(49)	1.00		1.00		225(47)	1.00		1.00	
Hypertriglyceridemia(200–268)	518(42)	0.79	0.47–1.34	0.81	0.47–1.40	223(49)	0.93	0.54–1.61	1.01	0.58–1.77
Upper normal (103–199)	1884(182)	0.93	0.64–1.36	0.99	0.65–1.52	2760(498)	0.99	0.67–1.45	1.07	0.72–1.58
Lower normal (≤102)	1881(187)	0.99	0.67–1.46	0.96	0.65–1.44	2819(412)	1.13	0.76–1.67	1.24	0.83–1.86
**HDL- cholesterol(mg/dl)** b										
Upper normal (≥51)	1803(186)	1.36	0.92–2.00	1.32	0.87–1.99	2713(400)	1.10	0.76–1.57	1.17	0.81–1.68
Lower normal (40–50)	1837(185)	1.49	1.01–2.20	1.43	0.96–2.13	2653(490)	1.19	0.84–1.69	1.23	0.86–1.76
Hypoalphalipoproteinemia(35–40)	522(39)	0.78	0.46–1.31	0.75	0.44–1.27	356(53)	0.77	0.48–1.26	0.76	0.47–1.25
Hypoalphalipoproteinemia (≤35)	632(50)	1.00		1.00		305(63)	1.00		1.00	
**Glucose(mg/dl)**										
Diabetes (≥126)	167(16)	1.00		1.00		86(13)	1.00		1.00	
Impaired fast glucose (110–125)	296(25)	0.64	0.28–1.45	0.63	0.27–1.46	191(40)	1.65	0.75–3.64	1.78	0.80–3.98
Normal (≤109)	4331(419)	1.21	0.61–2.39	1.22	0.61–2.43	5750(953)	2.05	1.02–4.14	2.24	1.10–4.55
**Smoking**										
Smoker	2291(189)	1.00		1.00		375(910)	1.00		1.00	
Past history of smoking	1618(192)	1.02	0.79–1.32	1.08	0.83–1.41	329(47)	0.92	1.60	0.95	0.55–1.64
Non-smoker	885(79)	1.15	0.82–1.61	1.16	0.82–1.64	5323(910)	0.98	0.64–1.50	1.00	0.65–1.53

Logistic regression analysis. Odds ratios (OR) and 95% confidence intervals (CI).

a Subjects with no history of cardiovascular disease, cerebrovascular disease or intake of medication for hyperlipemia, hypertension or diabetes.

b Following international standards, comparison between the upper half of the high risk group and the upper and lower halves of the normal group.

c Adjusted for age, education, household income, occupation, BMI, alcohol drinking and exercise habits.

The subjects with normal systolic blood pressure(≤79) had a higher incidence of cLBP than those in the upper half of the group(>95 mmHg) with diastolic blood pressure above 90 mmHg(the age adjusted OR was 2.12 in men and 1.61 in women). The estimates were little affected by the addition of covariates in the model. There was no relationship between systolic blood pressure, total cholesterol, TG, HDL, or smoking with chronic LBP in either men or women. For a more contrastive comparison, we divided the abnormal value group into 2 groups and compared the most extreme measurement group with normal ranges.

## Discussion

Our study found that the risk of chronic LBP increased in both men and women who had a history of cardiovascular diseases. But the Framingham risk score which predicts the danger of cardiovascular disease and almost all major cardiovascular risk factors were not associated with chronic LBP. The study sample was representative of Korean men and women aged 20 to 89 years. Our results are consistent with previous studies that reported cardiovascular diseases are associated with chronic LBP. Several studies have reported that, similar to our study results, cardiovascular diseases are related to LBP [11], [18] and lumbar disc degeneration[19]–[23] using the atherosclerosis hypothesis.

It cannot be stated that cardiovascular disease risk factors are associated with chronic LBP just on the basis that cardiovascular diseases and vessel abnormality are related with chronic LBP. In our study results, although there was a strong connection between chronic LBP and cardiovascular diseases, no consistent associations were found between chronic LBP and the major or “traditional” risk factors, especially the major Independent Risk Factors for CHD [14], which include serum total cholesterol, HDL cholesterol, triglycerides, diabetes mellitus and smoking. The association of these risk factors with chronic LBP has been a matter of controversy in many studies. High cholesterol [24], [25], high LDL cholesterol [24], [25], low HDL cholesterol [26], high triglycerides [24], [25], [27], diabetes [28] and hypertension [28], [29] have been found to be significantly and independently associated with LBP.

A systematic review by Shiri et al. [1] on cardiovascular and lifestyle risk factors and the relationship with lumbar radicular pain (sciatica) found obesity, a long smoking history, and serum C-reactive protein to be associated with sciatica, whereas no consistent associations between sciatica and serum lipid levels or high blood pressure were found. Another study reported that there was no association between diabetes and LBP [30].

Contrary to our assumption that hypertension patients would have a higher incidence of LBP, diastolic blood pressure was shown to have a negative correlation with LBP in both men and women. These results may result from lower susceptibility to musculoskeletal pain (ex. LBP) in people with high blood pressure [31]. Adverse to our initial prediction, the diabetic group presented a lower prevalence rate of low back pain when compared to the normal group. This may possibly be an error due to the small diabetic group.

Some major strengths of our study are that the sample group is a large-scale, nationally representative sample of Koreans, and the health examination and questionnaire surveys were conducted by trained interviewers in a standardized manner. Moreover, we analyzed not only the association between each major risk factor of cardiovascular disease and chronic LBP, but also whether cardiovascular risk factors could successfully predict chronic LBP using the Framingham risk score, and in this analyses, we were able to control for a number of potential confounders of chronic LBP, such as age, gender, BMI, household income, occupation, education, exercise, and alcohol drinking habits.

Two nationally representative sample studies on the relationship between LBP and cardiovascular disease risk factors have been published recently, and they are each based on the Finnish [32] and Norwegian population [27]. Leino-Arjas et al. reported that serum total cholesterol, LDL cholesterol and triglyceride levels had a significant association with sciatica in the Finnish population, and Heuch et al. reported that HDL cholesterol and triglycerides were related to LBP in the Norwegian population, which are not consistent with our results. This contrast between nationalities may result from the difference in dependent variables (sciatica vs. chronic LBP) or possibly the ethnic and cultural environmental diversities between Caucasians and Asians. A vast body of studies show across various ethnicities and cultures that relatively high risk factors of associated diseases are not necessarily reflected in the incidence rate [33], [34] and that occurrence rates within similar disease groups demonstrate racial differences [35].

Also, our study revealed that Koreans are relatively healthy. The incidence rate of chronic LBP of ≥3 months was 10.8% in Korean men and 21.1% in women, whereas it was 21% in Norwegian men and 26% in women. Koreans showed better health statistics in the anthropometric risk factor indices than Finnish and Norwegian populations too and this may have partially caused the racial differences in the association with cardiovascular risk factors. The fact that other studies did not consider cerebrovascular diseases occurring in relation with atherosclerosis may have relatively influenced the results as well.

Furthermore, 8-hour fasting prior to laboratory examination was conducted in our study to achieve higher accuracy in the cholesterol and triglyceride measures. However, the Finnish population underwent 4-hour fasting, and the Norwegian population were not regulated for the amount of fasting, but were adjusted for covariates statistically instead.

The limitations of the present study are as follows; first, the cross-sectional design of this study does not allow for definite conclusions be drawn about the causal relationship; second, the hypothesis that atherosclerosis of lumbar arteries may lead to disc degeneration, which may, in turn, cause chronic LBP or sciatica in people with cardiovascular disease or its risk factors is a weak point in that the low back region is infamous for its asymptomatic abnormalities [36].

Accordingly, even if the lumbar intervertebral disc or surrounding structures happen to be damaged or altered by atherosclerosis, there may be an absence of chronic LBP symptoms, rendering it difficult to affirm their correlation.

Also, we did not recognize the possibility of a direct correlation between cardiovascular disease and LBP, and this may be at variance with the atherosclerosis hypothesis proposed in this study. Additional limitations are that a major risk factor of cardiovascular disease – LDL cholesterol – was not included as a variable in our study, and that a minimum 8 hour fast may not have been enough to achieve completely reliable blood results regarding TG.

The reason why almost all major cardiovascular risk factors were not associated with chronic LBP in our study could be because of low statistical power due to the limited number of cases in the data or other potential biases due to the cross-sectional design nature of the study. These possibilities set aside, the results of our study can be largely interpreted from two different standpoints. Firstly, cLBP may be associated with psychological and/or sensitivity factors of cardiovascular disease patients unrelated to the atherosclerosis hypothesis. Individuals who reported having suffered from cardiac infarction or angina may be more sensitive and susceptible to pain in general. Although recent studies using national data from Finnish [32] and Norwegian populations [27] also did not consider psychological factors, as cLBP is closely related with psychological conditions, the potential relationship should be given due consideration.

Secondly, risk factors that are not necessarily “traditional” risk factors but related to the atherosclerosis hypothesis may be associated with chronic LBP in Koreans. For many years, serum cholesterol level has been viewed to be the single most significant biochemical marker in predicting the risk of cardiovascular disease, but it has since showed limitations as a preventive measure; i.e., failing to prevent cardiovascular disease even after successful “primary prevention” through reduction of risk factors [37]. Therefore more studies are focusing on novel serum markers, including C-reactive protein and homocysteine [38], as cardiovascular disease also occurs in many people with normal lipidemia levels and traditional risk factors were unable to predict the occurrence of cardiovascular disease in these patients. Also, Briggs et al. reported in a recent study that within abnormal BMI groups(BMI>30), patients with elevated C-reactive protein had a higher LBP occurrence rate with a higher OR of 2.87(95% CI 1.18–6.96) [26], [38]. Along these lines, perhaps a more significant connection may be drawn between chronic LBP in Koreans and a new cardiovascular risk factor.

In conclusion, our study indicates a strong relationship between chronic LBP and cardiovascular diseases. However, we were unable to confirm the cause of this relationship. Therefore, further large-scale studies on the association between various cardiovascular risk factors (such as high-sensitivity C-reactive protein) and chronic LBP considering psychological factors are called for.
